# Photovoltaic Microhabitats Reorganize Vegetation and Soil Carbon Pools in an Alpine Dryland Grassland

**DOI:** 10.3390/biology15151286

**Published:** 2026-08-04

**Authors:** Li Yan, Guangchao Cao, Jinrong Hu, Yan Wang

**Affiliations:** 1 Qinghai Provincial Key Laboratory of Physical Geography and Environmental Process, College of Geographical Sciences, Qinghai Normal University, Xining 810008, China; yll863890331@163.com (L.Y.); hujinrong_qhnu@163.com (J.H.); wx628412@163.com (Y.W.); 2 Key Laboratory of Qinghai–Tibet Plateau Land Surface Processes and Ecological Conservation (Ministry of Education), College of Geographical Sciences, Qinghai Normal University, Xining 810008, China; 3 Academy of Plateau Science and Sustainability, People’s Government of Qinghai Province and Beijing Normal University, Xining 810008, China

**Keywords:** photovoltaic microhabitats, carbon-pool structure, vegetation carbon pools, soil organic carbon stock, alpine dryland grassland

## Abstract

Utility-scale ground-mounted photovoltaic development is expanding across alpine dryland grasslands, creating engineered microhabitats with contrasting shading regimes, surface exposure, and soil hydro-physical conditions. Microhabitat-specific responses of vegetation and soil carbon pools remain poorly quantified in these systems. We assessed carbon-pool responses in the Talatan photovoltaic park on the northeastern Qinghai–Tibet Plateau using 109 plot-level observations across five microhabitats: reference grassland (REF), fixed-panel shaded microhabitat (FS), fixed-panel interspace microhabitat (FI), horizontal single-axis tracking microhabitat (HSA), and tilted single-axis tracking microhabitat (TSA). We estimated aboveground biomass carbon (AGB-C), belowground biomass carbon (BGB-C), 0–30 cm soil organic carbon stock (SOC stock), and total ecosystem carbon storage (TEC). Microhabitat contrasts were evaluated relative to REF, and standardized association models were used to examine relationships between SOC stock, soil moisture, soil fines, and vegetation carbon pools. Carbon responses differed by microhabitat position and carbon-pool compartment. FS showed the clearest vegetation carbon contrast, with AGB-C 29.2% lower than REF and BGB-C 29.8% lower with borderline statistical support. In contrast, FS showed smaller, more uncertain contrasts for SOC stock (−3.2%) and TEC (−6.3%). The SOC stock association model explained 46% of the variance, with positive coefficients for soil moisture (β = 0.496), soil fines (β = 0.256), AGB-C (β = 0.239), and BGB-C (β = 0.171). These findings indicate that photovoltaic carbon assessment in alpine dryland grasslands should distinguish microhabitat position, carbon-pool compartment, and soil hydro-physical background to identify where carbon responses occur and through which carbon pools they are expressed.

## Simple Summary

Large photovoltaic solar parks create engineered microhabitats beneath fixed panels, between panel rows, and within tracking systems. In the Talatan photovoltaic park on the northeastern Qinghai–Tibet Plateau, these microhabitats showed distinct vegetation and soil carbon-pool patterns. The clearest contrast occurred beneath fixed panels, where aboveground biomass carbon was about 29% lower than in nearby reference grassland and belowground biomass carbon showed a similar negative contrast. Soil organic carbon and measured total ecosystem carbon storage responded more weakly, consistent with the larger size and slower turnover of the soil carbon pool. Soil moisture and fine particles were closely linked with soil carbon storage, indicating that local soil background is important for interpreting vegetation–soil carbon linkages. These results suggest that carbon monitoring in alpine dryland photovoltaic grasslands should separate shaded, interspace, and tracking-system positions and assess vegetation and soil carbon pools separately.

## 1. Introduction

Solar energy development is increasingly reshaping terrestrial land systems while contributing to climate change mitigation. Ground-mounted photovoltaic (PV) parks occupy extensive land surfaces, and their development has become a land-system change process as well as an energy-transition strategy [1,2,3]. This issue is especially relevant in dryland, semi-arid grassland, and high-radiation regions, where high solar-resource availability often coincides with water limitation, sparse vegetation, fragile soils, and slow ecological recovery [4,5,6,7]. The climate benefit of PV generation is expressed mainly through avoided fossil-carbon emissions, while the ecological effect of ground-mounted PV infrastructure is expressed through changes in vegetation production, soil hydro-physical conditions, nutrient cycling, and plant–soil carbon processes on occupied land [6,8,9,10]. Compared with general land-cover change, PV land occupation has a stronger engineered structural component. Regularly arranged panels, supports, and array spacing continuously reshape near-surface light and heat conditions [10,11,12], as well as rainfall delivery and surface-water redistribution [13,14], creating spatially differentiated ecological response units across an originally continuous grassland surface. Based on this structural feature, ecological carbon assessment in grassland PV parks needs to examine how carbon-pool patterns are reorganized within the PV footprint.

This spatial differentiation mainly arises from the way PV panels reshape light, water, and heat processes near the ground. Panel shading reduces incoming radiation and modifies surface temperature and evaporative conditions [10,11,12]; panel interception and runoff concentration redistribute water inputs toward specific positions such as drip lines, panel edges, and interspaces [13,14]. As a result, beneath-panel zones, inter-panel spaces, drip-affected positions, and tracking-system corridors form short-distance differences in light exposure, shade duration, soil moisture, and near-surface thermal regimes [10,11,12,14,15]. Fixed-panel systems usually generate relatively stable shade and drip-line patterns, while single-axis tracking systems create temporally moving shade and different windows of surface exposure [11,15]. These differences change where plant growth conditions and soil processes occur inside solar arrays. In water-limited grasslands, moderate shading may reduce evaporative stress and improve vegetation growth conditions [4,6,15]; persistent shading, construction disturbance, surface alteration, and incomplete vegetation recovery may also constrain plant production and soil ecological functioning [8,16,17]. PV effects on grassland carbon pools have a clear positional structure and need to be identified at the microhabitat scale.

Microhabitat-scale environmental differences are expressed through different carbon pools. Aboveground biomass carbon (AGB-C) reflects standing shoot production, while belowground biomass carbon (BGB-C) reflects root biomass and potential root-derived carbon inputs [18,19]. Both pools usually respond rapidly to changes in light, water conditions, and local growing space. Soil organic carbon (SOC) stock integrates longer-term organic matter inputs, decomposition, mineral association, aggregate protection, and historical soil development, with a relatively slower rate of change [19,20,21,22]. In grassland ecosystems, SOC commonly accounts for the largest share of measured ecosystem carbon storage, especially when the assessment includes the upper soil layer [18,19,23]. When AGB-C, BGB-C, and SOC stock are directly combined into one aggregate carbon metric, the large SOC pool may dilute sensitive vegetation carbon differences among PV microhabitats. At the same time, analyzing vegetation carbon pools alone cannot determine whether these rapid changes are already associated with soil carbon storage. Separating vegetation carbon pools, SOC stock, and measured total ecosystem carbon storage within the quantified compartments can more clearly identify which carbon pool carries the PV-related carbon signal.

Previous studies have shown that PV parks can modify near-surface microclimate and soil moisture [10,11,12], vegetation structure and restoration trajectories [5,6,15,16], soil properties and soil ecological processes [8,17,24], and grassland carbon cycling and soil carbon storage [10,15,17,20,21]. A key question remains insufficiently resolved: engineered microhabitat position, carbon-pool compartment, installation type, and soil hydro-physical background are often discussed separately, with limited integration in a unified plot-scale carbon assessment. This limits our ability to determine three issues: where carbon responses occur inside PV arrays, whether these responses are carried by vegetation carbon pools or soil carbon pools, and whether SOC stock is regulated by soil moisture and fine-particle conditions. This issue is especially important in alpine dryland grasslands, where vegetation may respond rapidly to local light and soil-water changes [4,6,11,15], while SOC stock is more strongly controlled by soil texture, moisture conditions, root-derived inputs, and long-term organic matter stabilization processes [19,20,21,22,25]. Establishing a microhabitat-scale, pool-resolved, and soil-context-constrained carbon assessment framework can more accurately identify the spatial sources and process implications of ecological carbon effects inside grassland PV parks.

Here, we used the Talatan photovoltaic park on the northeastern Qinghai–Tibet Plateau as a field setting to examine carbon-pool patterns across reference grassland, fixed-panel microhabitats, and single-axis tracking microhabitats. Using 109 plot-level observations, we quantified vegetation carbon pools, 0–30 cm SOC stock, and measured total ecosystem carbon storage (TEC), and combined reference-grassland contrasts with standardized carbon-pool association models. We focused on three questions: (i) how carbon-pool structure varies across engineered photovoltaic microhabitats; (ii) how vegetation carbon pools and 0–30 cm SOC stock differ in their microhabitat contrasts; and (iii) how SOC stock is associated with soil moisture, soil fines, and vegetation carbon pools. By linking engineered microhabitat position, carbon-pool compartment, and soil hydro-physical background within a single empirical framework, this study provides a basis for microhabitat-resolved, pool-resolved, and soil-context-aware carbon assessment in alpine dryland photovoltaic grasslands.

## 2. Materials and Methods

### 2.1. Study Area

**Figure 1:**
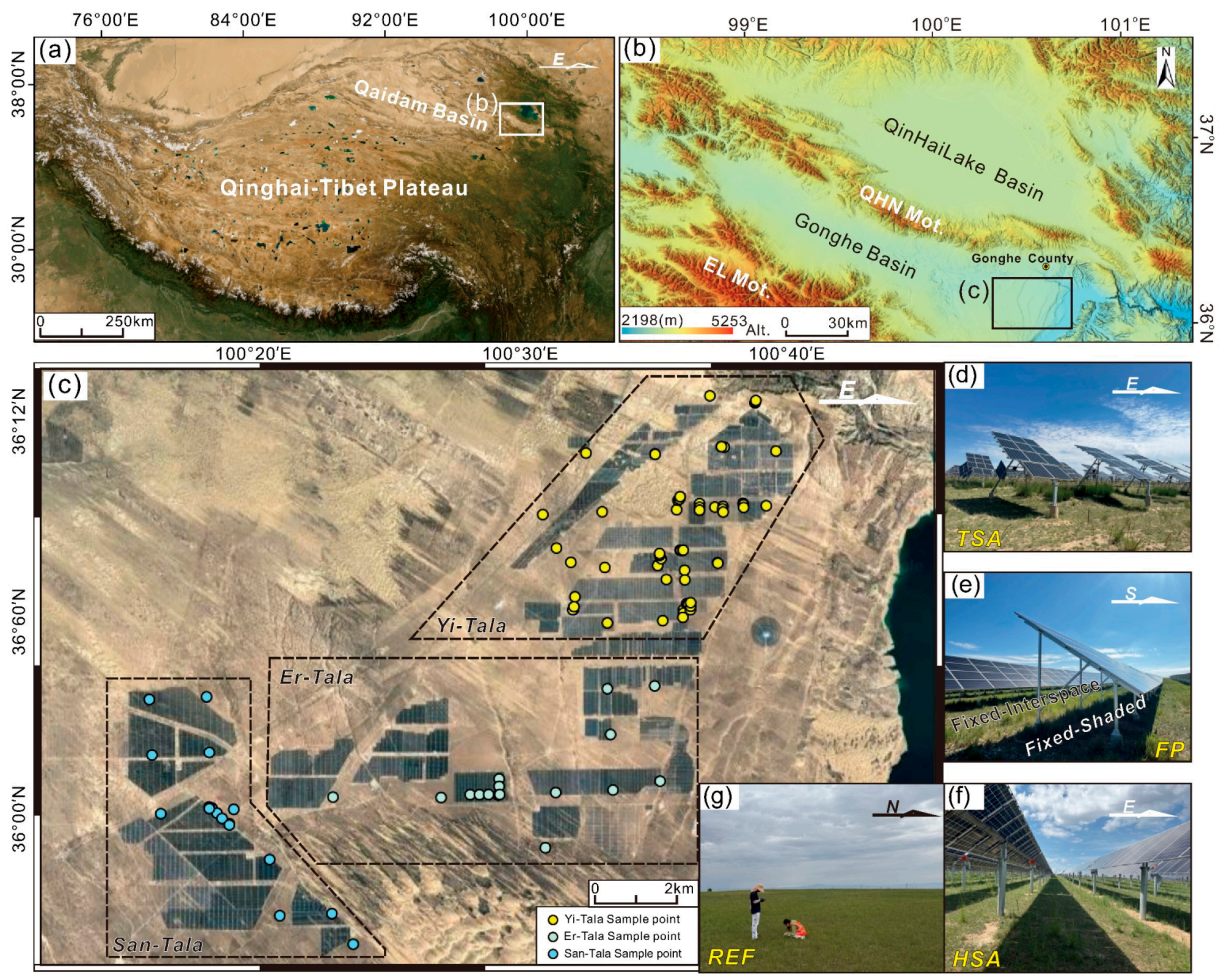
Study area, sampling distribution, and representative photovoltaic microhabitats in the Talatan photovoltaic park. (**a**) Location of the study region on the Qinghai–Tibet Plateau. (**b**) Topographic setting of the Gonghe Basin and surrounding mountain–basin systems, with the rectangle indicating the location of the Talatan photovoltaic park. (**c**) Satellite image showing the distribution of sampling points across the Yi-Tala, Er-Tala, and San-Tala subregions. Point colors denote sampling subregions rather than microhabitat classes, and dashed polygons indicate the main photovoltaic sampling areas. (**d**–**g**) Field photographs of representative microhabitats: tilted single-axis tracking microhabitat (TSA), fixed-panel microhabitats showing the fixed-panel shaded position (FS) and fixed-panel interspace position (FI), horizontal single-axis tracking microhabitat (HSA), and reference grassland (REF).

The study was conducted in the Talatan photovoltaic park (99°45′–100°30′ E, 36°00′–36°30′ N), located in Gonghe County, Hainan Tibetan Autonomous Prefecture, Qinghai Province, China, on the northeastern Qinghai–Tibet Plateau (Figure 1). The region lies at an elevation of approximately 2900–3100 m and has developed into a major utility-scale photovoltaic base in an alpine dryland environment.

The region has an alpine continental arid climate, with a mean annual temperature of 4.1 °C, mean annual precipitation of 246.3 mm, and mean annual evaporation of 1716.7 mm. Annual sunshine duration ranges from 2300 to 3500 h. The parent materials are mainly loess and gravel deposits, and soils are dominated by chestnut soils with sandy loam to light loam textures. Natural vegetation is sparse desert-steppe vegetation with a relatively simple community structure. Large-scale photovoltaic development began in 2013, and the park occupied approximately 609 km^2^ at the time of this study. Both fixed-panel and tracking photovoltaic systems occur within the park, creating distinct photovoltaic microhabitats that differ in shading regime and near-surface environmental conditions [25]. This heterogeneous infrastructure provides a suitable field setting for evaluating microhabitat-specific carbon-pool patterns in an alpine dryland photovoltaic grassland.

### 2.2. Microhabitat Classification and Field Sampling

Field sampling was conducted during the peak growing season in late July 2023. Based on photovoltaic infrastructure configuration and plot position relative to photovoltaic panels, sampling plots were classified into five microhabitats: reference grassland (REF), fixed-panel shaded microhabitat (FS), fixed-panel interspace microhabitat (FI), horizontal single-axis tracking microhabitat (HSA), and tilted single-axis tracking microhabitat (TSA) (Figure 1). REF plots were established in reference grassland approximately 1 km away from direct panel-covered areas to reduce potential panel-induced microclimatic influence while maintaining a comparable regional environmental background. FS plots were established beneath fixed photovoltaic panels, FI plots were located between adjacent fixed-panel rows, and HSA and TSA plots were located within horizontal and tilted single-axis tracking systems, respectively. Within each microhabitat, plots were selected to represent the dominant ground conditions of that microhabitat while avoiding roads, maintenance tracks, panel foundations, panel-row margins, and visibly disturbed patches. Each plot was treated as a plot-level replicate for the carbon-pool analyses. A total of 109 plots were surveyed, including 25 REF, 24 FS, 23 FI, 25 HSA, and 12 TSA plots. The 609 km^2^ park area describes the overall scale of the photovoltaic landscape, whereas plot allocation followed a stratified microhabitat-comparison design rather than area-proportional sampling. Replication was kept approximately balanced among accessible microhabitat classes where feasible. The smaller number of TSA plots reflected the limited deployment area and lower availability of suitable tilted single-axis tracking sites within the accessible sampling region. A 50 cm × 50 cm quadrat was established within each plot for aboveground biomass harvest, and belowground biomass and soil samples were collected within the same plot in alignment with the quadrat layout.

Aboveground biomass was harvested by clipping all standing vegetation at ground level within each quadrat. Belowground biomass was collected from the 0–30 cm soil profile using a soil auger with a diameter of 7 cm, with sampling aligned with the 0–10, 10–20, and 20–30 cm depth intervals. Root samples were separated from soil material, washed, oven-dried at 65 °C to constant mass, summed across the three depth intervals, and converted to dry biomass per unit area using the auger cross-sectional area.

Soil samples were collected from fixed-depth intervals of 0–10, 10–20, and 20–30 cm. Fixed-depth sampling was used to ensure comparability of 0–30 cm carbon-stock estimates across microhabitats; soil genetic horizons were not separately delineated. At each depth interval, three subsamples were obtained along the quadrat diagonal and combined into one composite sample for soil organic carbon and particle-size analyses. Undisturbed soil cores were collected with cutting rings at the corresponding depths for soil moisture and bulk density measurements. Visible roots, stones, and plant residues were removed before laboratory analyses. The field sampling yielded 324 depth-layer soil samples from 109 plots across the three planned depth intervals. Two plots had incomplete 0–30 cm soil profiles because abundant coarse stones prevented auger and cutting-ring insertion into the 10–20 or 20–30 cm layers. Complete 0–30 cm soil profiles were therefore available for 107 plots. SOC stock and TEC calculations were based on these 107 complete profiles, whereas aboveground and belowground biomass carbon analyses used all 109 plots. Plot-level vegetation and soil measurements were subsequently integrated for carbon-pool analyses.

### 2.3. Laboratory Measurements and Carbon-Pool Calculation

Soil moisture content and bulk density were measured using undisturbed soil cores collected with 100 cm^3^ cutting rings at each depth interval. Soil moisture content was determined gravimetrically by oven-drying fresh undisturbed cores at 105 °C to constant mass. Bulk density was calculated as oven-dried soil mass divided by core volume. Soil particle-size distribution was measured using a Mastersizer 3000 laser diffraction particle-size analyzer (Malvern Panalytical Ltd., Malvern, UK) and classified according to the USDA particle-size classification [26]. Clay was defined as particles <2 μm, silt as particles of 2–50 μm, and sand as particles of 50–2000 μm. Soil fines content was calculated as the sum of the clay and silt fractions, corresponding to particles <50 μm. Soil samples were acidified to remove inorganic carbon before SOC measurement, and soil organic carbon concentration was then determined using a Vario TOC cube total organic carbon analyzer (Elementar, Germany). For association analyses, 0–30 cm soil moisture content and soil fines content were calculated as thickness-equivalent means across the 0–10, 10–20, and 20–30 cm layers.

The aboveground and belowground biomass carbon pools were estimated from dry biomass using a plant carbon fraction of 0.47, following the default biomass carbon fraction recommended in IPCC carbon-stock accounting guidelines [23]. Because aboveground and belowground biomass had been converted to areal biomass density (g m^−2^), vegetation carbon pools were calculated as:

(1) $$C_{veg}=B\times f_{c}/100$$

where $C_{veg}$ is the vegetation carbon pool (Mg C ha^−1^), B is dry biomass density (g m^−2^), and $f_{c}$ is the plant carbon fraction. The same conversion was applied to aboveground and belowground biomass. Sensitivity analyses using plant carbon fractions of 0.45 and 0.50 were conducted to evaluate the robustness of vegetation carbon-pool estimates.

Soil organic carbon stock was calculated for each soil layer following standard soil carbon-stock accounting [23]:

(2) $$SOC_{stock}=SOC\times BD\times D\times 0.1$$

where $SOC_{stock}$ is soil organic carbon stock (Mg C ha^−1^), SOC is soil organic carbon concentration (g kg^−1^), BD is bulk density (g cm^−3^), and D is soil layer thickness (cm). Soil organic carbon stocks from the 0–10, 10–20, and 20–30 cm layers were summed to obtain the 0–30 cm soil organic carbon stock for each plot. Because gravel content was not quantified, SOC stock was calculated without coarse-fragment correction and should be interpreted as a coarse-fragment-uncorrected 0–30 cm SOC stock estimate.

Measured total ecosystem carbon storage (TEC) was calculated as the sum of the carbon pools quantified in this study:

(3) $$TEC=C_{AGB}+C_{BGB}+SOC_{0-30}$$

where TEC is the measured total ecosystem carbon storage within the quantified aboveground biomass, 0–30 cm belowground biomass, and 0–30 cm SOC pools (Mg C ha^−1^), $C_{AGB}$ is AGB-C, $C_{BGB}$ is BGB-C, and $SOC_{0-30}$ is the 0–30 cm soil stock.

### 2.4. Statistical Analysis

All statistical analyses were conducted at the plot level. The response variables were the aboveground biomass carbon pool (AGB-C), belowground biomass carbon pool (BGB-C), 0–30 cm soil organic carbon stock (SOC stock), and the measured total ecosystem carbon storage (TEC), defined as the sum of AGB-C, BGB-C, and 0–30 cm SOC stock. Analyses were performed in Python 3.9 using pandas, NumPy, SciPy, and statsmodels. Statistical tests were two-sided, and *p* < 0.05 was used as the nominal significance threshold before false-discovery-rate correction.

The primary analysis used ordinary least-squares linear models with photovoltaic microhabitat as a categorical predictor and reference grassland (REF) as the reference category:

(4) $$Y_{i}=\beta_{0}+\sum_{m}\beta_{m}I(M_{i}=m)+\varepsilon_{i}$$

where $Y_{i}$ is the response variable for plot i, $\beta_{0}$ represents the mean value of REF, and $\beta_{m}$ represents the contrast between each photovoltaic microhabitat and REF. Microhabitat differences were evaluated from the model-estimated contrasts relative to REF.

To reduce the influence of extreme observations, response variables were winsorized at the 2.5th and 97.5th percentiles prior to model fitting [27]. For each response variable, observations below the 2.5th percentile and above the 97.5th percentile were replaced by the corresponding percentile values. This procedure modified six observations for each response variable, with three observations at each tail. Heteroscedasticity-consistent HC3 standard errors were used for coefficient tests and statistical inference [28,29]. Effect sizes were expressed as percentage changes relative to REF. Bootstrap 95% confidence intervals were obtained by resampling plots with replacement and refitting the corresponding models 2000 times.

To account for multiple microhabitat testing, *p*-values were adjusted using the Benjamini–Hochberg false discovery rate procedure [30].

Robustness analyses were conducted to evaluate the stability of the primary microhabitat contrasts. These analyses retained the same plot-level linear-model framework and included: (i) region-adjusted models incorporating sampling region as an additional covariate; (ii) deployment-time-adjusted sensitivity models incorporating the available field-recorded deployment-time variable as a standardized continuous covariate; (iii) sensitivity analyses using alternative plant carbon fractions of 0.45 and 0.50; (iv) a no-TSA sensitivity analysis to assess whether the FS contrasts depended on retaining the smaller TSA group; and (v) comparisons and correlation analyses between 0 and 30 cm SOC concentration and 0–30 cm SOC stock. In the deployment-time-adjusted models, the deployment-time variable was z-standardized within the model dataset before fitting. These models were used only to test whether the primary microhabitat contrasts were robust to adjustment for the available deployment-time information, rather than to estimate an independent temporal effect of photovoltaic operation duration.

### 2.5. Carbon-Pool Association Analysis

To evaluate statistical associations among photovoltaic microhabitats, vegetation carbon pools, soil hydro-physical conditions, and 0–30 cm SOC stock, we constructed a set of standardized multiple linear regression models.

In the association models, both response variables and continuous explanatory variables were z-standardized before model fitting; categorical microhabitat coefficients therefore represent standardized differences relative to REF. Three association models were evaluated. The first model examined variation in AGB-C as a function of photovoltaic microhabitat, 0–30 cm soil moisture content, and 0–30 cm soil fines content. The second model used the same explanatory variables to evaluate variation in BGB-C.

The third model evaluated associations with 0–30 cm SOC stock. In addition to photovoltaic microhabitat, soil moisture content, and soil fines content, AGB-C and BGB-C were included as explanatory variables to assess whether standing vegetation carbon pools were associated with SOC stock at the plot level.

Bulk density was not included in the primary association models because 0–30 cm SOC stock was calculated using bulk density. Including bulk density as an explanatory variable would introduce structural dependence between predictors and the SOC stock used as the response variable. Models including bulk density were evaluated only as robustness analyses.

All association models were fitted using ordinary least-squares regression with HC3 heteroscedasticity-consistent standard errors. Standardized regression coefficients, 95% confidence intervals, and two-sided *p*-values were used to evaluate the direction, uncertainty, and relative strength of statistical associations. These analyses were intended to characterize carbon-pool association patterns rather than to provide a complete predictive model or evidence of causal pathways.

## 3. Results

### 3.1. Carbon-Pool Structure Differed Among Photovoltaic Microhabitats

To describe the carbon-pool structure across photovoltaic microhabitats, we compared aboveground biomass carbon, belowground biomass carbon, 0–30 cm soil organic carbon stock, and measured total ecosystem carbon storage among REF, FS, FI, HSA, and TSA. Across all microhabitats, vegetation carbon pools were much smaller than soil organic carbon stock, with mean AGB-C ranging from 0.196 to 0.323 Mg C ha^−1^ and mean BGB-C ranging from 1.266 to 2.667 Mg C ha^−1^.

**Figure 2:**
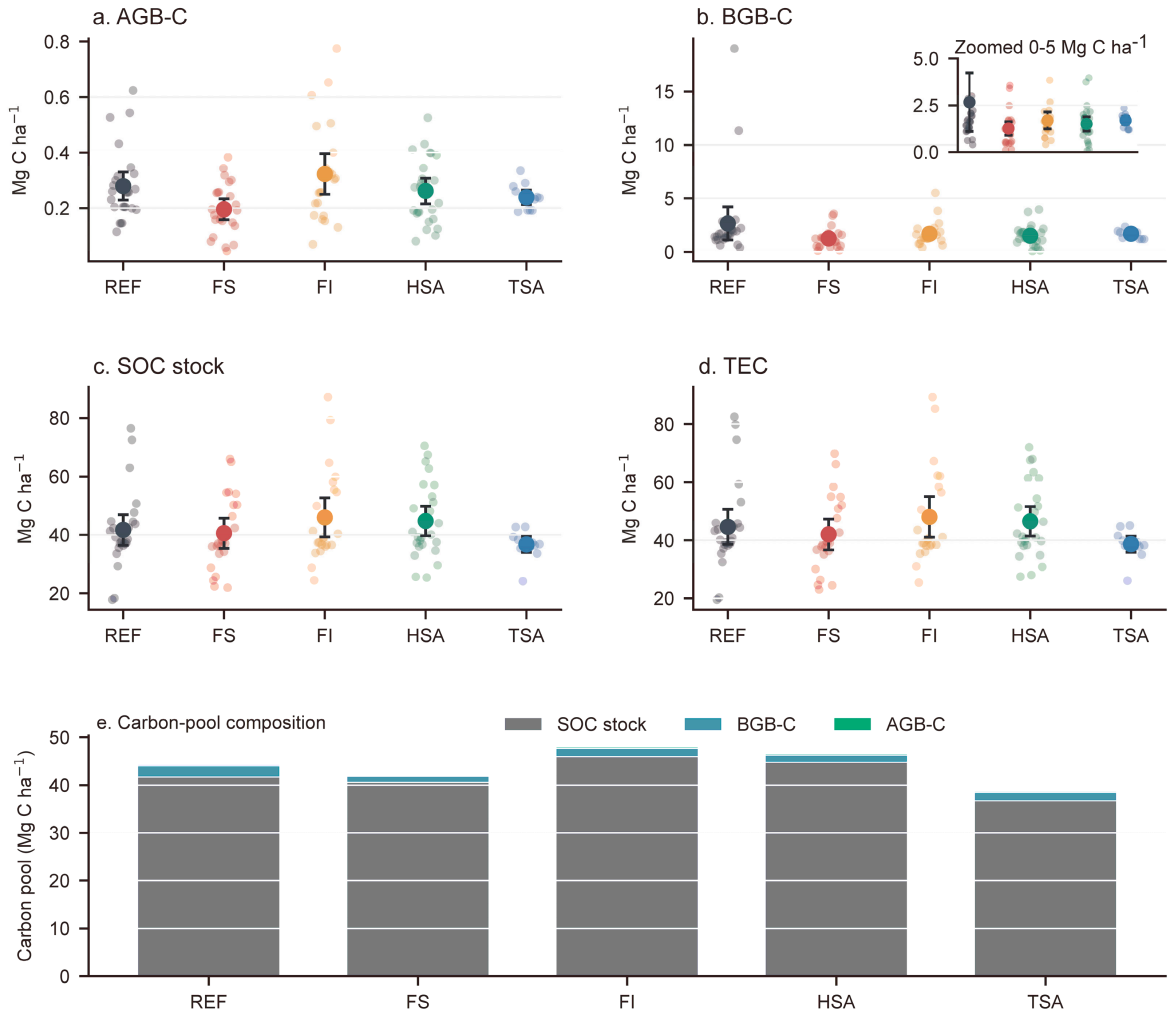
Carbon-pool structure across photovoltaic microhabitats in an alpine dryland grassland. (**a**) Aboveground biomass carbon pool (AGB-C); (**b**) belowground biomass carbon pool (BGB-C); (**c**) 0–30 cm soil organic carbon stock (SOC stock); (**d**) measured total ecosystem carbon storage (TEC); and (**e**) carbon-pool composition across reference grassland (REF), fixed-panel shaded microhabitat (FS), fixed-panel interspace microhabitat (FI), horizontal single-axis tracking microhabitat (HSA), and tilted single-axis tracking microhabitat (TSA). Points represent individual sampling plots. Large circles indicate group means and error bars denote 95% confidence intervals. In panel (**b**), the inset shows the 0–5 Mg C ha^−1^ range to facilitate visualization of the main distribution. Panel (**e**) presents mean carbon-pool composition for each microhabitat. Carbon pools are expressed as Mg C ha^−1^.

For AGB-C, the highest mean value occurred in FI (0.323 Mg C ha^−1^), whereas the lowest value occurred in FS (0.196 Mg C ha^−1^; Figure 2a). For BGB-C, REF had the highest mean value (2.667 Mg C ha^−1^), whereas FS had the lowest mean value (1.266 Mg C ha^−1^; Figure 2b). Mean SOC stock ranged from 36.737 Mg C ha^−1^ in TSA to 45.953 Mg C ha^−1^ in FI, and FS (40.568 Mg C ha^−1^) was close to REF (41.687 Mg C ha^−1^; Figure 2c). A similar pattern was observed for TEC, with mean values ranging from 38.667 Mg C ha^−1^ in TSA to 48.014 Mg C ha^−1^ in FI (Figure 2d).

The stacked carbon-pool composition showed that SOC stock accounted for most of TEC in all five microhabitats, whereas AGB-C contributed only a small fraction of the measured total carbon pool (Figure 2e). Together, these descriptive patterns indicated that carbon-pool composition differed among microhabitats, with stronger relative variation in vegetation carbon pools than in the dominant soil carbon pool.

### 3.2. Fixed-Panel Shaded Microhabitat Showed the Clearest Negative Vegetation Carbon Contrast

**Figure 3:**
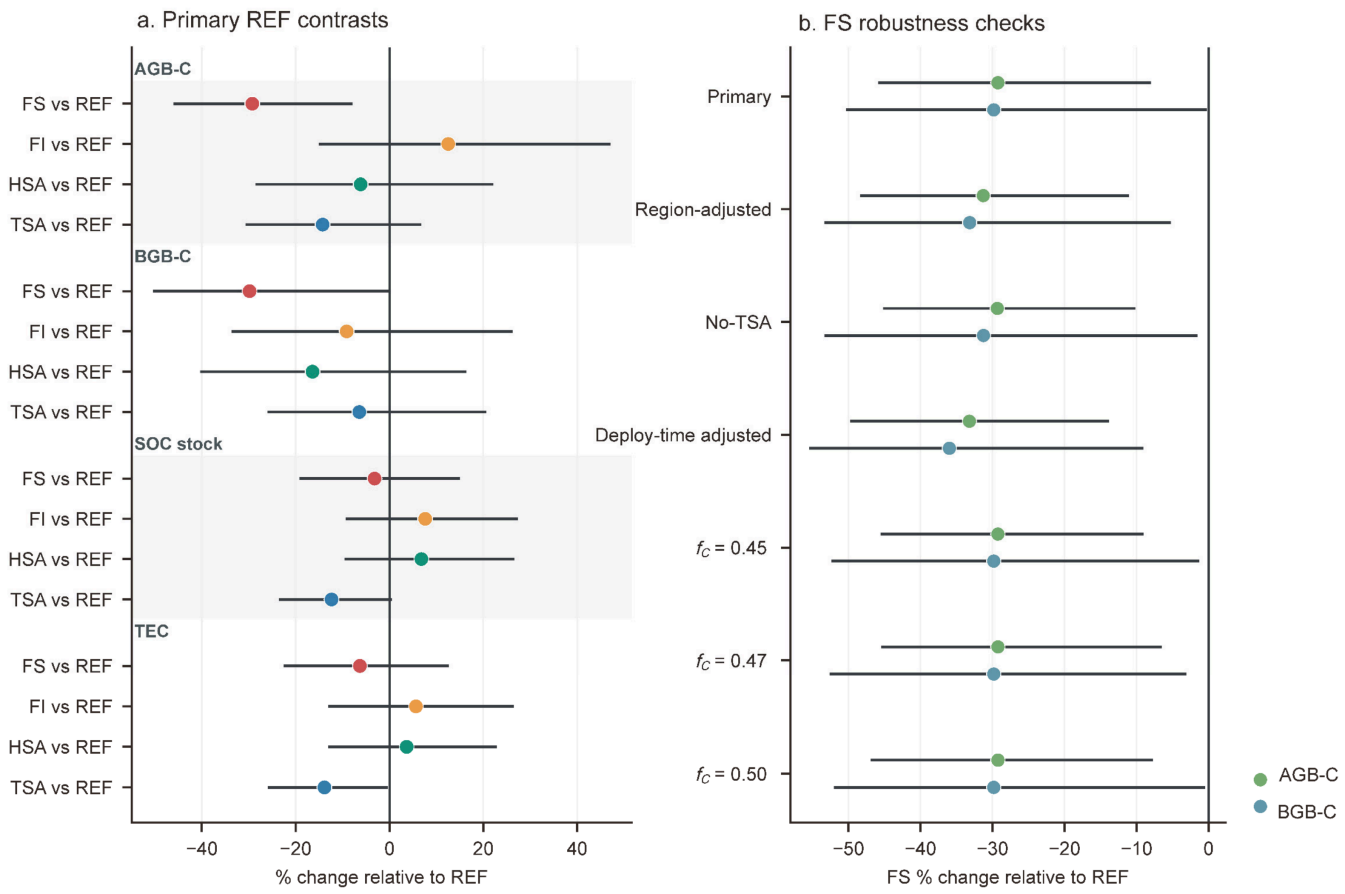
Reference-grassland contrasts and robustness analyses for vegetation carbon pools in photovoltaic microhabitats. (**a**) Percentage changes in aboveground biomass carbon pool (AGB-C), belowground biomass carbon pool (BGB-C), 0–30 cm soil organic carbon stock (SOC stock), and total ecosystem carbon storage (TEC) relative to reference grassland (REF). Points represent estimated contrasts and horizontal bars indicate bootstrap 95% confidence intervals. (**b**) Robustness analyses for the fixed-panel shaded microhabitat (FS). Percentage changes in AGB-C and BGB-C relative to REF are shown for the primary model, region-adjusted model, no-TSA sensitivity analysis, deployment-time-adjusted model, and alternative plant carbon fractions (*f_C_* = 0.45, 0.47, and 0.50). Points represent estimated contrasts and horizontal bars indicate bootstrap 95% confidence intervals.

To quantify microhabitat contrasts relative to the reference grassland, we fitted plot-level linear models for each carbon-pool variable using REF as the reference category. In the primary model, FS showed the largest negative AGB-C contrast relative to REF, with a reduction of 29.2% (bootstrap 95% CI: −45.7% to −8.2%; *p* = 0.0115; FDR-adjusted *p* = 0.0461; Figure 3a). FS also showed a negative BGB-C contrast relative to REF, with a reduction of 29.8% and nominally borderline support before FDR correction (bootstrap 95% CI: −50.1% to −0.4%; *p* = 0.0505; FDR-adjusted *p* = 0.2020; Figure 3a). By contrast, the FS contrasts for SOC stock and TEC were smaller and more uncertain, with estimated changes of −3.2% and −6.3%, respectively, and confidence intervals crossing zero. The remaining photovoltaic microhabitats did not show a comparable negative pattern across both AGB-C and BGB-C in the primary REF contrasts.

Robustness analyses supported the stability of the FS vegetation carbon contrasts. After adding sampling region as a covariate, FS remained negative for both AGB-C (−31.3%) and BGB-C (−33.2%; Figure 3b). The no-TSA sensitivity analysis produced similar FS contrasts for AGB-C (−29.3%) and BGB-C (−31.2%), indicating that the FS vegetation carbon pattern did not depend on retaining the smaller TSA group. Deployment-time-adjusted sensitivity models also preserved negative FS contrasts for AGB-C (−33.2%) and BGB-C (−36.0%). Because the plant carbon fraction scaled all biomass values proportionally, alternative carbon fractions of 0.45, 0.47, and 0.50 yielded the same relative FS contrast for AGB-C (−29.2%) and preserved the ranking of microhabitat vegetation carbon pools, with FS remaining the lowest microhabitat for both AGB-C and BGB-C. Across the primary and robustness analyses, FS consistently showed the strongest negative vegetation carbon contrast among the photovoltaic microhabitats.

### 3.3. Vegetation Carbon Pools Showed Stronger Relative Responses than SOC Stock

**Figure 4:**
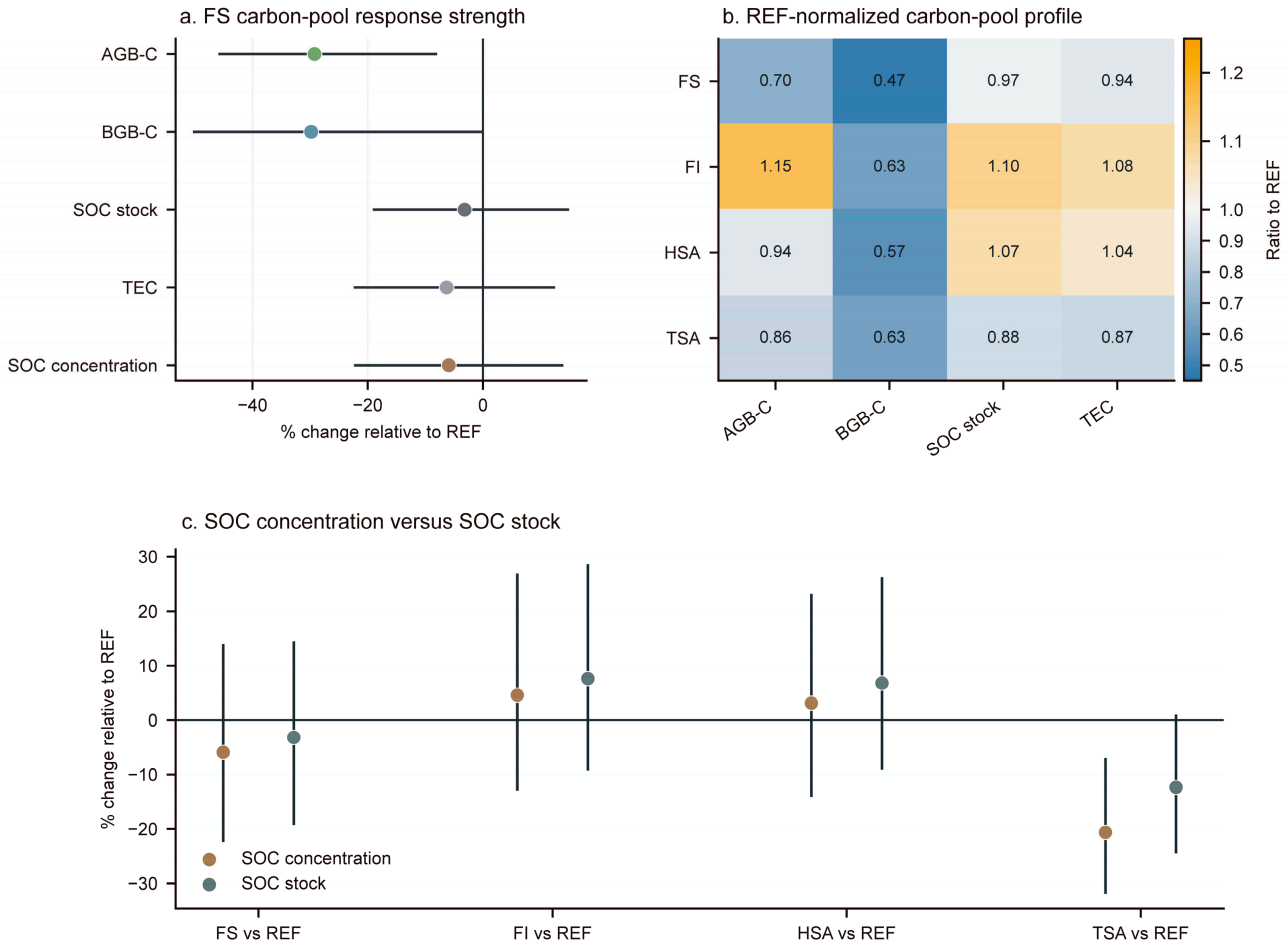
Relative vegetation carbon and SOC responses across photovoltaic microhabitats. (**a**) Percentage changes in AGB-C, BGB-C, 0–30 cm SOC concentration, 0–30 cm SOC stock, and TEC in the fixed-panel shaded microhabitat (FS) relative to reference grassland (REF). Points represent estimated contrasts and horizontal bars indicate bootstrap 95% confidence intervals. (**b**) REF-normalized carbon-pool profiles for AGB-C, BGB-C, 0–30 cm SOC stock, and TEC across photovoltaic microhabitats. Values represent the ratio of the microhabitat mean to the REF mean. (**c**) Comparison between responses based on 0–30 cm SOC concentration and 0–30 cm SOC stock across photovoltaic microhabitats. Points represent estimated percentage changes relative to REF and error bars denote bootstrap 95% confidence intervals.

To compare relative response magnitudes among carbon pools, we examined model-estimated REF contrasts and REF-normalized carbon-pool profiles for vegetation carbon pools, 0–30 cm SOC stock, and TEC. Within FS, the model-estimated negative contrasts for AGB-C (−29.2%) and BGB-C (−29.8%) were larger in magnitude than those for SOC stock (−3.2%) and TEC (−6.3%) (Figure 4a). These contrasts indicated larger relative vegetation carbon responses than SOC stock responses in FS.

REF-normalized carbon-pool profiles further showed that FS had lower relative values for AGB-C (0.70) and BGB-C (0.47), whereas SOC stock (0.97) and TEC (0.94) remained close to REF (Figure 4b). This profile showed that the largest relative departure from REF occurred in vegetation carbon pools, especially BGB-C, whereas SOC stock remained closer to REF. FI and HSA showed REF-normalized SOC stock values greater than 1.0, whereas TSA showed lower relative values across most carbon-pool indicators (Figure 4b).

Comparisons based on 0–30 cm SOC concentration and 0–30 cm SOC stock yielded consistent directional responses across all microhabitats (Figure 4c). For example, FS and TSA showed negative contrasts under both metrics, whereas FI and HSA showed positive contrasts under both metrics. Across plots, 0–30 cm SOC concentration and 0–30 cm SOC stock were strongly correlated (Pearson r = 0.985, *p* < 0.001; Spearman ρ = 0.971, *p* < 0.001; *n* = 107), confirming that concentration-based and stock-based SOC metrics showed consistent plot-level variation. Together, these results indicate that vegetation carbon pools exhibited larger relative microhabitat-related variation than SOC stock.

### 3.4. Carbon-Pool Association Analyses Revealed Distinct Vegetation- and Soil-Carbon Linkages

**Figure 5:**
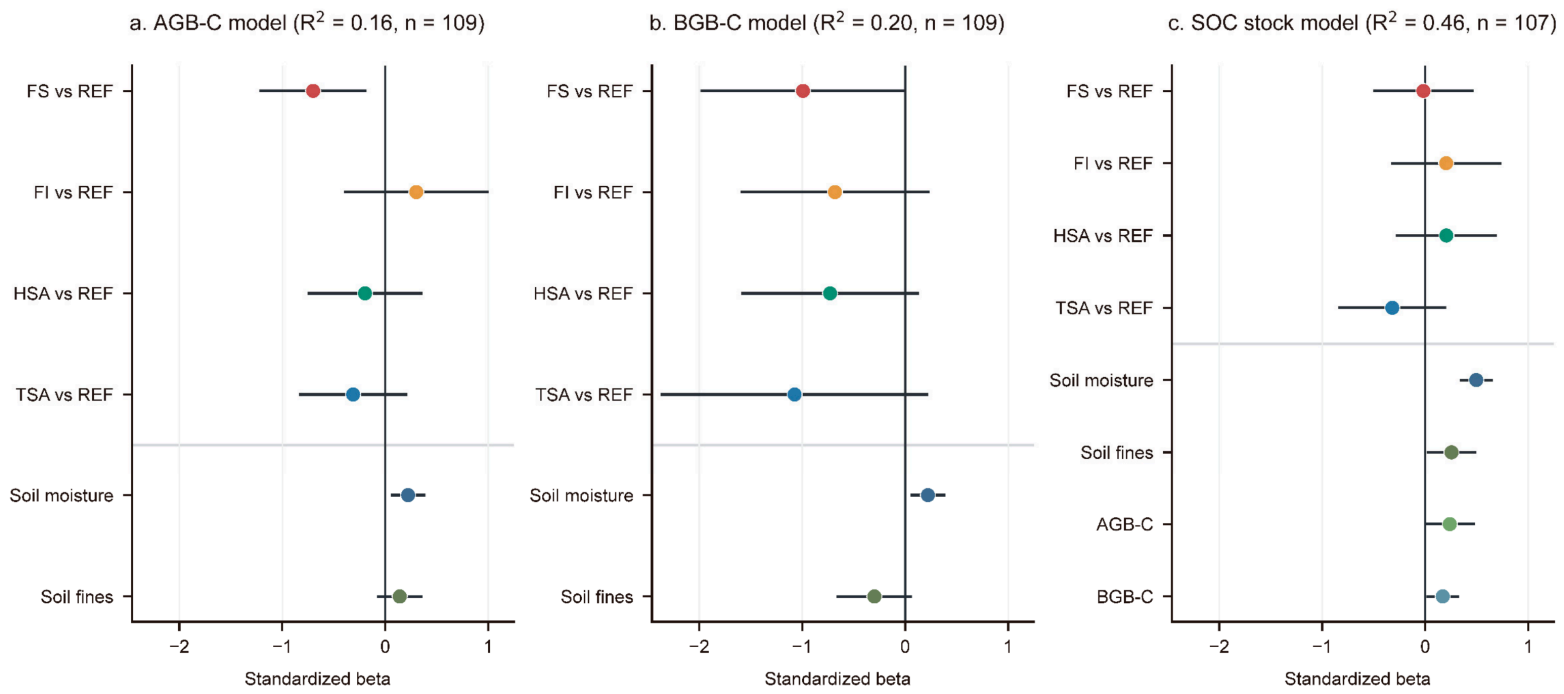
Standardized carbon-pool association models across photovoltaic microhabitats. (**a**) Standardized multiple-regression model for aboveground biomass carbon pool (AGB-C). (**b**) Standardized multiple-regression model for belowground biomass carbon pool (BGB-C). (**c**) Standardized multiple-regression model for 0–30 cm soil organic carbon stock (SOC stock). Points represent standardized regression coefficients (β), and horizontal bars indicate 95% confidence intervals. Response variables and continuous explanatory variables were z-standardized before model fitting. REF was used as the reference category for microhabitat contrasts. Model coefficients are shown for photovoltaic microhabitats, soil moisture content (0–30 cm), soil fines content (0–30 cm), and vegetation carbon pools where applicable. The coefficient of determination (R^2^) and sample size (*n*) are reported for each model.

To characterize statistical associations among photovoltaic microhabitats, vegetation carbon pools, soil hydro-physical variables, and 0–30 cm SOC stock, we fitted standardized multiple regression models for AGB-C, BGB-C, and SOC stock. In the AGB-C model (R^2^ = 0.16, *n* = 109), FS remained negatively associated with AGB-C after accounting for soil moisture and soil fines, with a standardized coefficient of β = −0.700 (95% CI: −1.207 to −0.194; *p* = 0.0068; Figure 5a). Soil moisture and soil fines showed positive coefficients in the AGB-C model. In the BGB-C model (R^2^ = 0.20, *n* = 109), FS also remained negatively associated with BGB-C, with a standardized coefficient of β = −0.993 (95% CI: −1.975 to −0.011; *p* = 0.0475; Figure 5b). The soil-moisture and soil-fines coefficients had the same directions as those in the AGB-C model.

The SOC stock model explained a larger proportion of variance (R^2^ = 0.46, *n* = 107) than the vegetation carbon models (Figure 5c). Within this model, soil moisture showed the strongest positive coefficient for SOC stock (β = 0.496), followed by soil fines (β = 0.256). AGB-C (β = 0.239) and BGB-C (β = 0.171) also showed positive coefficients for SOC stock. By contrast, the microhabitat coefficients in the SOC stock model were smaller than those observed in the vegetation carbon models. Together, these association analyses indicated that vegetation carbon pools had clearer microhabitat-related associations, whereas SOC stock was more closely associated with soil moisture, soil fines, and vegetation carbon pools.

## 4. Discussion

### 4.1. Microhabitat-Resolved Assessment Identifies Within-Park Carbon-Pool Heterogeneity

Resolving ground-mounted photovoltaic arrays into engineered microhabitats provides a clearer basis for interpreting within-park carbon-pool heterogeneity. Panel position, shading stability, rainfall redistribution, surface exposure, and tracking mode can generate distinct near-surface environments within the same solar park, including differences in light availability, soil hydrothermal conditions, and vegetation processes [10,11,14,31]. Evidence from the Gonghe Basin further shows that large photovoltaic power stations can modify microclimatic conditions in desert environments, providing regional support for interpreting near-surface environmental heterogeneity in the Talatan photovoltaic landscape [32]. Consistent with this microhabitat perspective, the reference plots and four photovoltaic microhabitats in the Talatan photovoltaic park showed different carbon-pool profiles (Figure 2).

The central implication is that within-park photovoltaic heterogeneity can be missed when carbon assessment relies only on aggregated PV-versus-reference or total-carbon comparisons. Because SOC stock dominated the measured carbon pools, aggregate TEC patterns were more strongly influenced by the soil carbon pool than by the smaller vegetation carbon pools. This scaling effect helps explain why vegetation carbon pools provided clearer microhabitat contrasts than 0–30 cm SOC stock or TEC. This pattern indicates that aggregate carbon metrics can obscure localized vegetation carbon contrasts when soil carbon dominates the measured ecosystem carbon pool.

This pattern supports a shift from simple PV-versus-reference comparisons toward an array-internal interpretation of ecological heterogeneity. In a utility-scale solar park, beneath-panel, interspace, tracking-system, and reference positions are not interchangeable sampling or monitoring units. They represent different combinations of shade duration, open-sky exposure, surface disturbance, and water redistribution. Treating these positions separately allows carbon-pool contrasts to be interpreted in relation to the physical organization of PV infrastructure. It also connects carbon accounting with ecological interpretation, because the same aggregate carbon value can arise from different combinations of AGB-C, BGB-C, and 0–30 cm SOC stock. Recent photovoltaic carbon and soil-ecological studies have similarly emphasized that installation type, site context, vegetation management, and soil-carbon pathways should be considered in photovoltaic carbon assessment [8,10,16,20,21].

In this alpine dryland grassland, the main discussion point is which microhabitat and which carbon-pool compartment carried the clearest contrast. AGB-C and BGB-C represent standing vegetation carbon pools and near-term carbon-input potential, whereas SOC stock integrates longer-term organic matter accumulation, decomposition, stabilization, and soil development [22,33]. Therefore, identifying which carbon pool shows the clearest contrast is essential for interpreting photovoltaic carbon patterns without over-relying on aggregate carbon metrics.

### 4.2. Fixed-Panel Shaded Microhabitats Showed the Clearest Vegetation Carbon Constraint

The clearest vegetation carbon contrast occurred in the fixed-panel shaded microhabitat. Relative to reference grassland, FS showed lower AGB-C and a similar negative BGB-C contrast, although the latter had borderline statistical support. The FS vegetation carbon contrast was also retained in region-adjusted, no-TSA, deployment-time-adjusted, and plant-carbon-fraction sensitivity analyses. These results support treating FS as the main localized vegetation carbon constraint within the photovoltaic park.

This pattern is consistent with the physical structure of fixed-panel shaded positions. Fixed panels repeatedly cover the same ground area, creating persistent shading and potentially modifying near-surface thermal and moisture conditions [4,10,11,16]. Panel cover can also redistribute rainfall through sheltering, runoff concentration, and drip-line effects, potentially altering where and when water reaches the soil surface within arrays [13,14]. In alpine dryland grasslands, where vegetation growth is constrained by short growing seasons, sparse cover, and water limitation, such small-scale changes in light and water supply may be reflected in standing vegetation carbon pools [11,34]. The observed FS contrast is also consistent with previous Talatan evidence showing lower aboveground and belowground biomass in under-module fixed-axis plots than in control and tracking-system plots [25]. Compared with the stronger aboveground-biomass contrast reported in a temperate grassland solar park, where aboveground biomass in gap and control areas was approximately four times that under PV arrays [10], the Talatan FS response represents a moderate but consistent vegetation carbon constraint.

The ecological importance of the FS contrast lies in the spatial persistence of the fixed-panel footprint. In tracking systems, shading and direct radiation generally move across the ground surface during the day, spreading exposure across a larger area. In contrast, fixed panels tend to impose recurring light and rainfall redistribution conditions on the same beneath-panel patches [14]. In water-limited alpine grasslands, this spatially stable modification may influence shoot production and root development through changes in light availability, soil hydrothermal conditions, and water redistribution. This interpretation is supported by solar-park studies showing that panel cover can alter ground-level microclimate, soil temperature, soil moisture, vegetation biomass, photosynthesis, and plant–soil carbon processes [10,11,16]. The observed reduction in standing vegetation carbon under FS can therefore be interpreted as a plausible carbon-pool expression of a persistent fixed-panel microenvironment, although this pathway remains inferential because photosynthetically active radiation, soil temperature, soil moisture, and rainfall redistribution were not directly monitored in this study.

The presence of a negative BGB-C contrast alongside lower AGB-C is ecologically relevant because it links the FS pattern not only to standing shoot carbon but also to the standing root carbon pool. Lower AGB-C is consistent with reduced standing shoot carbon, whereas lower BGB-C suggests a smaller standing root carbon pool. Because roots provide a major pathway for plant-derived carbon to enter soil through root turnover, rhizodeposition, and belowground litter, reduced BGB-C suggests weaker belowground carbon-input potential [18,19]. Direct measurements of root turnover, rhizodeposition, and root-derived carbon fluxes are needed to quantify the associated belowground carbon pathway.

The contrast between FS and other photovoltaic microhabitats further suggests that vegetation carbon responses may depend partly on the spatial persistence of panel cover. Fixed-panel interspaces remain more exposed to open-sky conditions, and tracking systems create moving shade rather than repeated cover over the same surface. This configuration-related interpretation is consistent with the physical distinction between fixed and single-axis tracking PV systems [35] and with evidence that PV panels can modify ground-level soil temperature and moisture conditions [11]. Longer-term observations from ground-mounted photovoltaic sites also show that panel cover can modify soil physical, chemical, and biochemical properties, reinforcing the need to distinguish beneath-panel positions from more exposed array spaces [17]. The TSA pattern should be interpreted cautiously because of its smaller sample size and more spatially limited deployment within the available samples. Overall, the evidence supports treating fixed-panel shaded positions as a priority microhabitat for detecting vegetation carbon constraints in this alpine dryland photovoltaic grassland.

### 4.3. Soil Hydro-Physical Background Helps Explain Weaker SOC-Stock Contrasts

The weaker SOC-stock contrast is consistent with the longer response time and stronger hydro-physical background dependence of soil carbon pools compared with standing vegetation carbon. Standing vegetation carbon is expected to be more sensitive to near-term changes in light, water availability, and near-surface microclimate, whereas SOC stock integrates organic matter inputs, microbial processing, decomposition, mineral association, aggregate protection, and historical soil development over longer time scales [22,33,36,37]. In this context, vegetation carbon reduction may be observed before a comparable change becomes detectable in the existing 0–30 cm SOC stock. The weak SOC-stock contrast observed here is smaller than the 9% lower SOC and the 23.6–29.1% lower particulate organic matter reported under solar panels in another temperate ground-mounted solar study [38], indicating that bulk SOC stock can show a more buffered response than labile or particulate carbon pools.

The association model is consistent with this interpretation by showing that SOC stock was more closely aligned with soil hydro-physical conditions and vegetation carbon pools than with microhabitat position alone. In the SOC-stock model, soil moisture showed the strongest positive association with SOC stock, followed by soil fines, AGB-C, and BGB-C (Figure 5). These associations suggest that SOC stock in the Talatan alpine dryland grassland was associated with water availability, fine-particle background, and plant carbon inputs. These association models should not be interpreted as complete process models; the modest explanatory power of the vegetation carbon models indicates that unmeasured factors, including photosynthetically active radiation, soil temperature, fine-scale rainfall redistribution, species composition, grazing or management history, and construction disturbance, may also have contributed to AGB-C and BGB-C variation.

Soil moisture can influence SOC dynamics through multiple pathways [37]. It may affect plant productivity and root growth, thereby influencing organic matter inputs [37], while moisture availability can also shape microbial activity, decomposition, and organic matter turnover [39]. In alpine dryland grasslands, where precipitation is limited and evaporative demand is high, small differences in soil-water retention may contribute to differences in biomass production and belowground carbon supply [11,34]. Soil texture provides another stabilizing context, represented in this study by soil fines. Silt and clay fractions increase surface area for organic matter association and support aggregate formation, both of which can enhance SOC stabilization [37,40]. In coarse-textured soils, spatial variation in fine-particle content may therefore contribute to differences in SOC preservation even when vegetation inputs are similar. These mechanisms provide a literature basis for interpreting why SOC stock in this study was associated not only with vegetation carbon pools, but also with moisture and fine-particle background [18,33,36,37,40]. This interpretation is consistent with previous Talatan work showing that soil moisture and texture were dominant drivers of nutrient spatial patterns under photovoltaic deployment [25].

Thus, the vegetation–soil asymmetry observed here is consistent with differences in carbon-pool sensitivity and response time. FS showed the clearest standing vegetation carbon contrast, whereas 0–30 cm SOC stock showed weaker contrasts, consistent with its larger pool size, slower turnover, and dependence on soil hydro-physical background. For photovoltaic carbon assessment, weak cross-sectional contrasts in 0–30 cm SOC stock should be interpreted together with vegetation carbon pools, soil moisture, fine-particle context, and SOC fractions to determine whether reduced vegetation carbon-input potential may translate into measurable soil-carbon change over longer monitoring periods.

### 4.4. Implications and Limitations for Microhabitat- and Pool-Resolved Carbon Monitoring

These findings support a microhabitat- and pool-resolved framework for carbon monitoring in alpine dryland photovoltaic grasslands. This framework is consistent with the broader view that multi-use solar landscapes require site-specific monitoring designs that account for ecosystem-service trade-offs, local environmental context, and management objectives [41]. Park-scale mean comparisons may merge shaded, interspace, tracking, and reference positions into a single average, masking the locations where carbon-pool contrasts are most clearly expressed. In the Talatan photovoltaic park, fixed-panel shaded positions should be treated as priority positions for monitoring vegetation carbon constraints.

This framework also has practical implications for sampling design. Future assessments of photovoltaic grasslands should separate shaded, interspace, and tracking-system positions as distinct sampling units. Fixed-panel shaded positions should be sampled separately from fixed-panel interspaces, and tracking-system microhabitats should be retained as comparative units for separating persistent-shade conditions from moving-shade conditions. Such a design would allow monitoring programs to identify whether carbon-pool contrasts are concentrated beneath fixed panels, distributed across array interspaces, or less pronounced under tracking systems. It would also help distinguish near-term vegetation carbon contrasts from slower SOC-stock changes. This distinction is important because recent photovoltaic grassland studies show that vegetation diversity, biomass, soil moisture, and soil physicochemical properties can respond jointly to large-scale PV deployment, and that grassland recovery under PV infrastructure may involve coupled water- and nutrient-use processes [6,15].

Future monitoring should be organized around three linked components. First, microclimate and hydrological redistribution should be measured using photosynthetically active radiation, soil temperature, rainfall redistribution, and continuous soil moisture observations to evaluate how fixed-panel structure modifies near-surface conditions. Second, vegetation carbon-input processes should be tracked through repeated AGB-C and BGB-C measurements together with observations of root turnover, rhizodeposition, and root-derived carbon fluxes. Third, soil carbon transformation should be evaluated using SOC stock, particulate organic carbon, and mineral-associated organic carbon to determine whether vegetation-input changes are expressed in labile plant-derived pools before becoming detectable in SOC stock [22,33]. This monitoring structure is consistent with growing evidence that photovoltaic facilities can influence vegetation communities, soil physicochemical properties, microbial communities, and nutrient cycling in dryland ecosystems [7,24].

The stock-based design of this study provides field-based evidence for microhabitat- and pool-specific carbon-pool contrasts in an alpine dryland grassland. It indicates where carbon-pool contrasts were expressed, which pools showed the clearest contrasts, and how soil hydro-physical background shaped vegetation–soil carbon linkages. Quantifying annual sequestration rates and carbon flux pathways will require repeated measurements and process observations. BGB-C should be interpreted as standing root biomass carbon rather than a direct belowground carbon flux, and because gravel content was not measured, 0–30 cm SOC stock should be interpreted as a coarse-fragment-uncorrected stock estimate. The single-park, cross-sectional design also means that broader inference requires repeated monitoring, balanced sampling of tracking-system microhabitats, and multi-site validation across comparable alpine dryland photovoltaic grasslands. Overall, photovoltaic carbon assessment in alpine dryland grasslands should move from park-scale average carbon comparisons toward identifying where carbon-pool contrasts emerge, through which carbon pools they are expressed, and over what timescale they become detectable and ecologically consequential.

## 5. Conclusions

This study indicates that photovoltaic microhabitats in the Talatan alpine dryland grassland were associated with microhabitat-specific and carbon-pool-specific patterns rather than a single uniform park-scale photovoltaic response. The clearest contrast was a vegetation carbon constraint in fixed-panel shaded microhabitats, where AGB-C was 29.2% lower than in reference grassland and BGB-C showed a similar negative contrast of −29.8%, although with borderline statistical support. In contrast, FI and HSA did not show the same consistent negative vegetation carbon pattern as FS, whereas TSA represented the lower end of the observed carbon-pool profile within the available tracking-system samples. These findings indicate that shaded, interspace, and tracking-system positions should be treated as distinct sampling and monitoring units when assessing carbon pools within large photovoltaic parks.

The results further indicate an asymmetry between standing vegetation carbon and soil carbon storage. In FS, the contrasts for 0–30 cm SOC stock and measured TEC were much weaker than those for vegetation carbon pools, with changes of −3.2% and −6.3%, respectively. This suggests that 0–30 cm SOC stock remained comparatively buffered at the plot scale, whereas vegetation carbon pools provided more sensitive near-term indicators of microhabitat constraints. The association analysis further showed that SOC stock was more closely linked to soil hydro-physical background, especially soil moisture (β = 0.496) and soil fines (β = 0.256), than to microhabitat position alone.

For monitoring and management, fixed-panel shaded positions should be treated as priority zones for detecting vegetation carbon constraints, while FI and HSA should be retained as comparative microhabitats for identifying less constrained or more buffered carbon-pool conditions. Although these single-park, observational, stock-based findings require validation through long-term monitoring, direct measurements of microclimate and carbon-input processes, and multi-site comparisons, they provide a field-based basis for microhabitat-resolved, pool-resolved, and soil-context-aware carbon assessment in alpine dryland photovoltaic parks.

## Data Availability

Data will be made available on request.
